# Heavy metal concentrations in floodplain soils of the Innerste River and in leaves of wild blackberries (*Rubus fruticosus* L. agg.) growing within and outside the floodplain: the legacy of historical mining activities in the Harz Mountains (Germany)

**DOI:** 10.1007/s11356-021-17320-w

**Published:** 2021-11-17

**Authors:** Louisa F. Steingräber, Catharina Ludolphy, Johannes Metz, Lars Germershausen, Horst Kierdorf, Uwe Kierdorf

**Affiliations:** 1 Department of Biology, University of Hildesheim, Universitätsplatz 1, 31141 Hildesheim, Germany; 2 Niedersächsischer Landesbetrieb für Wasserwirtschaft, Küsten- und Naturschutz, Betriebsstelle Hannover-Hildesheim, An der Scharlake 39, 31135 Hildesheim, Germany

**Keywords:** Biomonitoring, Floodplain, Harz Mountains, Heavy metals, Legacy pollution, Metal ore mining, *Rubus fruticosus* L. agg

## Abstract

We studied heavy metal levels in floodplain soils of the Innerste River in northern Germany and in the leaves of wild blackberries (*Rubus fruticosus* L. agg.) growing within and in adjacent areas outside the river floodplain. Heavy metal contamination of the Innerste floodplain is a legacy of historical metal ore mining, processing, and smelting in the Harz Mountains. The heavy metal (Cd, Pb, Zn, Cu, Ni, and Cr) contents of previously studied soil samples from eleven floodplain sites along the Innerste River were re-analyzed statistically, and the levels of these metals in blackberry leaves were determined at five sites. Mean concentrations in the floodplain soils were elevated by factors of 4.59 to 28.5 for Cd, 13.03 to 158.21 for Pb, 5.66 to 45.83 for Zn, and 1.1–14.81 for Cu relative to the precautionary limits for soils stipulated by the German Federal Soil Protection and Contaminated Sites Ordinance. Cadmium, Pb, Zn, Cu, and Ni levels in floodplain soils decreased markedly downstream, as did the concentrations of Cd, Zn, and Ni in the leaves of blackberries from within the floodplain. Levels of Cd, Pb, and Zn in leaves of blackberries from within the floodplain significantly exceeded those of specimens from outside the floodplain. The findings of our study highlight the potential of wild blackberry as a biomonitor of soil pollution by Cd, Pb, and Zn and corroborate the massive heavy metal contamination of floodplain soils along the Innerste River observed in previous studies.

## Introduction

Heavy metals are typically defined as metals with a density greater than 5 g/cm^3^ (Oves et al. 2012). They are naturally present in the Earth’s crust and cycle through the biogeosphere, but anthropogenic activities have greatly increased their release into the environment, where they are widely transported by wind and water (Kabata-Pendias and Mukherjee 2007). Heavy metals are non-biodegradable, undergo bioaccumulation and biomagnification (Ganesan 2012; Flache et al. 2016), and can pose significant health risks to humans (Kabata-Pendias and Mukherjee 2007; Ali et al. 2013), wildlife (Beyer et al. 2013; Wiemeyer et al. 2017), and entire ecosystems (Liu et al. 2019; Gorena et al. 2020). Some heavy metals, like cadmium (Cd) and lead (Pb), are nonessential and exhibit toxic effects even at low concentrations (Beyersmann and Hartwig 2008, Sigel et al. 2015, Joshi et al. 2019). Others, like copper (Cu), nickel (Ni), and zinc (Zn), have important physiological functions, yet are toxic above certain threshold values (Joshi et al. 2019). Therefore, knowledge of heavy metal levels in the environment is important for assessing metal-related ecological risks in an area (Nadgórska-Socha et al. 2017). Biomonitoring is frequently used to assess metal levels in the environment (Lin 2015). Vascular plants take up metals primarily (but not exclusively) from the soil via their roots, and plant biomonitoring can therefore provide a useful tool for geochemical risk assessment (Bianchini et al. 2012).

Numerous studies have addressed the bioavailability of heavy metals to plant species, and metal uptake and accumulation by plants growing on metalliferous soils around contaminated industrial and mining sites (Boularbah et al. 2006; Remon et al. 2013; Hu et al. 2014; Zhan et al. 2014; Favas et al. 2018; Wechtler et al. 2019; Nujkić et al. 2020). Positive relationships between heavy metal levels in soil and air and the concentrations found in plants growing at contaminated sites have been demonstrated (D’Souza et al. 2010, Al-Khashman et al. 2011; Galal and Shehata 2015). These relationships are often complex, as metal uptake and accumulation by plants depend on the chemical speciation and related bioavailability of the heavy metals, which in turn are affected by soil properties like pH, soil organic matter and cation exchange capacity, specifics of plant physiology, and phenology (Keane et al. 2001; Du Laing et al. 2009; Čurlík et al. 2016).

Several studies worldwide addressed the suitability of blackberries (*Rubus fruticosus* L. agg.) for biomonitoring or phytoremediation of contaminated areas (Baroni et al. 2004; Yoon et al. 2006; Reglero et al. 2008; Marques et al. 2009; Massa et al. 2010; Moreira et al. 2011; Nujkić et al. 2016). Blackberry shrubs are often found on railway and road embankments, allotments, alluvial landscapes, fallow land, and in light woodland or along forest edges. Blackberries are pseudophanerophytes with 2-year-old woody shoots (Ossig and Brandes 2019). Due to their chemical constituents (ascorbic acid and other organic acids, tannins, and essential oils), blackberries are a traditional herbal medicine (Verma et al. 2014; Vlad et al. 2019). The consumption of plant parts with elevated metal content may pose a health risk for humans and animals (National Research Council 2005, Kabata-Pendias and Mukherjee 2007).

Floodplains are transition zones between aquatic and terrestrial ecosystems, and the distribution of heavy metals in riverine and floodplain ecosystems varies due to different factors (Miller 1997; Besser et al. 2007; Hürkamp et al. 2009; Weber and Opp 2020). Fluvial transport of heavy metals occurs largely via suspended solids that are deposited in floodplains during flooding events (Zheng et al. 2008; Hürkamp et al. 2009; Parzych and Sobisz 2018). Floodplain soils therefore constitute long-term sinks for heavy metals, but can also become sources when heavy metals are remobilized during flooding events (Hürkamp et al. 2009).

Heavy metals released via runoff from tailings and slag heaps and those originating from atmospheric emissions due to smelting activities can contaminate soils and river sediments in the immediate vicinity of point sources as well as floodplains and river sediments further downstream (Miller 1997; Hürkamp et al. 2009; Ponting et al. 2021). The long-term release of heavy metals from metalliferous tailings or slag heaps constitutes a major environmental health risk for downstream areas. Due to climate-driven increase of extreme rainfall, flood events are expected to increase globally (Hilscherova et al. 2007; Ponting et al. 2021). The resulting changes in the flow regime of river systems, with more intense flooding, erosion, and drainage (Lynch et al. 2018), will increase the risk of heavy metal remobilization in floodplains (Hilscherova et al. 2007; Ponting et al. 2021).

The Harz Mountains in Northern Germany are rich in metalliferous minerals and have a long history of mining for silver (Ag), Cu, Pb, and Zn. The ores of the overburden that were mined in the Upper Harz area were mainly galena (PbS) and sphalerite (ZnS), and to a lesser extent also chalcopyrite (CuFeS_2_) (Deicke 2009). Mining activities in the Harz region first peaked between the twelfth and fourteenth centuries CE and then again between the fifteenth and the beginning of the twentieth century. Metal ore mining in the Harz Mountains ceased in 1992, when the last mine was closed. The long-standing mining, processing, and smelting of metal ores resulted in large amounts of harmful waste containing residual heavy metals (Deicke 2009). Mill tailings and the slag waste from smelting activities were deposited along several Harz rivers, including the Innerste River (Hellwig 2002). Heavy metals were transported into rivers due to contact of water with ore deposits, during ore processing, and by wash-out from the waste heaps (Ernst et al. 2009). These metals were transported downstream, and, in consequence, high contents of heavy metals, particularly Pb, Cd, Cu, and Zn, are present in sediments, floodplain soils, and vegetation along the Innerste River (Nowak and Preul 1971; Ernst et al. 2009; Knolle 2009). The high heavy metal concentrations in plants growing on the contaminated soils have repeatedly caused severe toxicosis in biota (Meyer 1822; Haarstick 1910; Knolle and Knolle 1983; Matschullat et al. 1997; Knolle et al. 2011).

The present study reports the concentrations of six heavy metals (Cd, chromium (Cr), Cu, Ni, Pb, and Zn) in floodplain soils along a section of the Innerste River and in leaves of wild blackberries growing within and outside the Innerste floodplain. Thus far, information on heavy metal levels in native spontaneous vegetation growing in the episodically inundated Innerste floodplain is lacking. Wild blackberry was selected as indicator species because of its widespread historic (Meyer 1822) and current natural occurrence throughout the study area. In this way, we assessed the suitability of wild blackberry as a biomonitor of heavy metal contamination. Specifically, we tested whether heavy metal concentrations of floodplain soils and in leaves of blackberries growing within the floodplain, and in adjacent regions outside the floodplain, decrease with distance from the Harz Mountains, the source region of the contamination.

## Materials and methods

### Study region

**Fig. 1:**
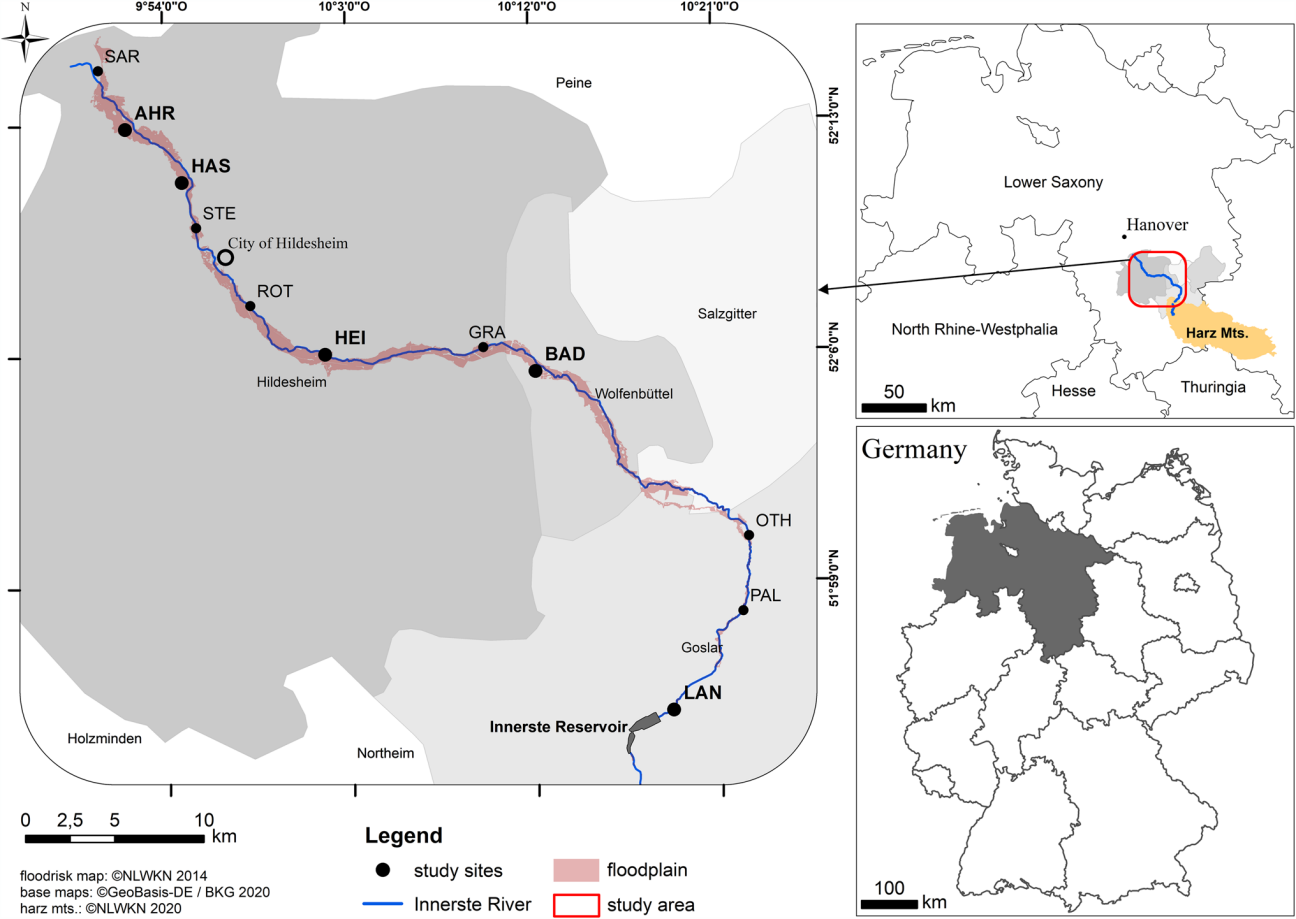
Location of the Harz Mountains and the study region in Germany and of the soil and plant sampling sites along the Innerste River in the counties of Hildesheim, Goslar, and Wolfenbüttel. Soil samples were collected at all eleven sites; leaf samples were collected at the five sites given in bold. Sites: **Langelsheim (LAN)**, Palandsmühle (PAL), Othfresen (OTH), **Baddeckenstedt (BAD)**, Grasdorf (GRA), **Heinde (HEI)**, Roter Stein (ROT), Steuerwald (STE), **Hasede (HAS)**, **Ahrbergen (AHR)**, and Sarstedt (SAR)

The Innerste River (51° 47′ to 52° 14′ N, 10° 22′ to 9° 49′ E) is located in the SE of the federal state of Lower Saxony (Germany) and runs mainly through the counties of Goslar and Hildesheim (Fig. 1). The study region has a subcontinental to subatlantic climate with an average annual precipitation of 683 mm and an average annual temperature of 8.7° C (Climate-Data.org 2020). The Innerste River originates in the Upper Harz Mountains south of Clausthal-Zellerfeld at about 600 m above sea level. After approximately 99 km, it flows into the Leine River. The Innerste has a catchment area of 1264 km^2^ along an altitude difference of about 540 m. From the upper to the lower reaches, the Innerste River flows through the natural regions of the Harz Mountains, the Weser-Leine Highlands, and the Hildesheim Börde in the northwestern Harz foreland. The upstream rock beds in the Upper Harz Mountains are composed of greywackes as well as siliceous and argillaceous shales (Liessmann 2010), while the downstream section is characterized by calcareous sediments from loess accumulations (Kroll 2005). At the “Heinde” water gauge, the average discharge (period 1953 − 2015) is 8.13 m^3^ × s^−1^ (NLWKN, 2018). Our study region starts 25 km downstream of the origin of the Innerste River, and below the outflow from the Innerste Reservoir (Fig. 1) near Langelsheim. Construction of the reservoir, which has a storage volume of 19.27 hm^3^, was completed in 1966. The reservoir is used for flood protection, low water elevation, and drinking water supply (Harzwasserwerke GmbH 2019). The last major flooding event, which caused inundation of nearly the entire Innerste floodplain and set some new records for high water marks, occurred in July 2017 (NLWKN, 2017).

### Study design, sample collection, and analysis

#### Soil

**Fig. 2:**
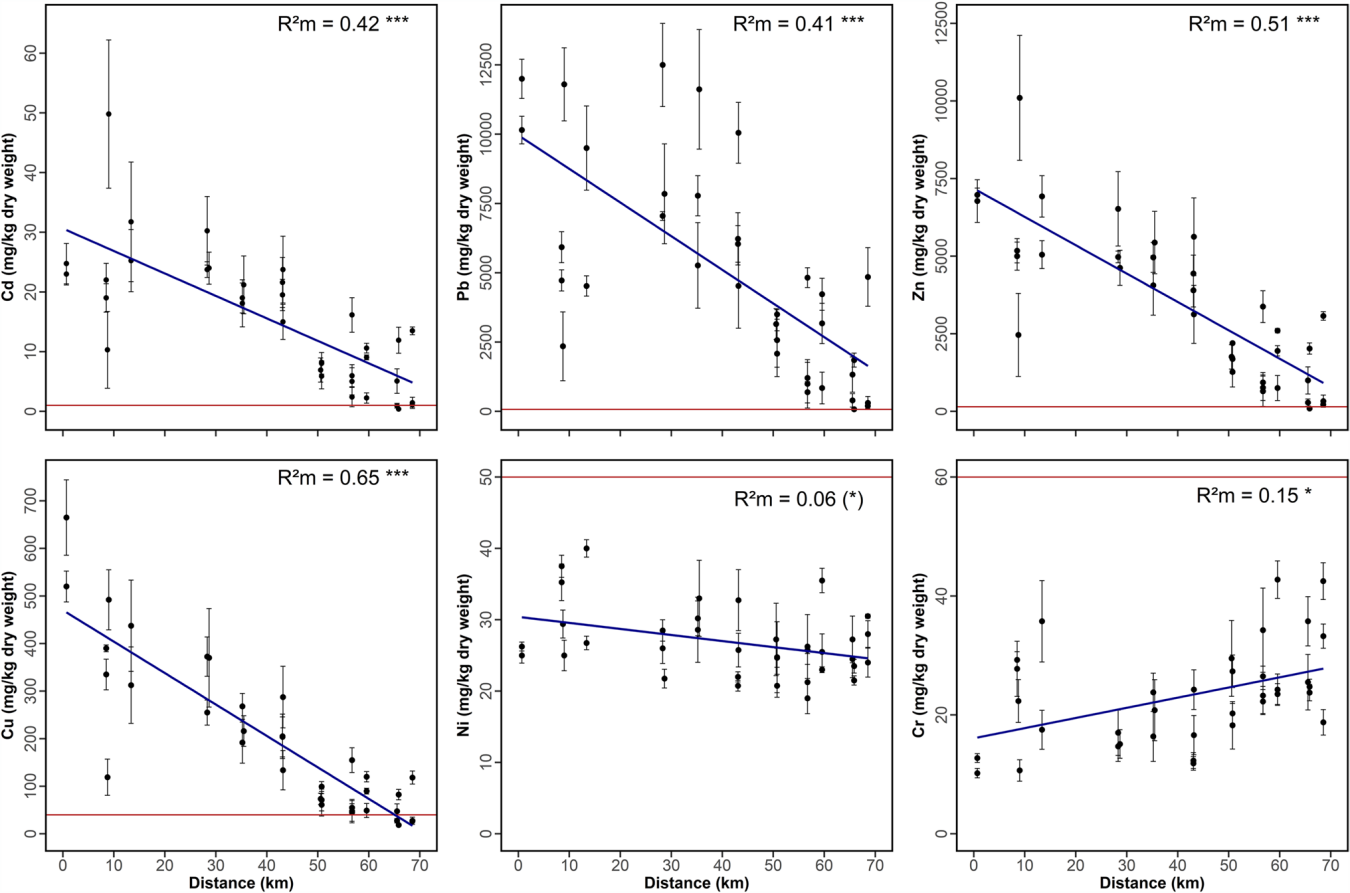
Mean (± SE) heavy metal content in 36 soil profiles from 11 sites along the Innerste River with increasing distance from the Innerste Reservoir. All soil profiles were located within the floodplain. The red horizontal lines indicate the precautionary limits for heavy metals according to the German Federal Soil Protection and Contaminated Sites Ordinance (BBodSchV, 1999). The significance of the effect “distance from the Innerste Reservoir” along with marginal *R*^2^ values (R^2^m) is added to ease interpretation (see Table 3 for full statistical results). Note the different scaling for each heavy metal. (*) 0.05 < *P* < 0.1, * *P* < 0.05, *** *P* < 0.001

To study the variation of heavy metal (Cd, Cr, Cu, Ni, Pb, and Zn) concentrations in floodplain soils with increasing distance from the outflow from the Innerste Reservoir (here used as a reference point), a total of 37 soil profiles had previously been recorded at eleven sites along the river in spring and summer of 2009 and 2010 (Table 1 and Fig. 1; Germershausen 2013). The soil samples had been digested according to DIN 38414, and heavy metal concentrations had been determined by a certified laboratory (Lower Saxony State Office for Water Management, Coastal Protection and Nature Conservation, NLWKN) using inductively coupled plasma optical emission spectroscopy (ICP-OES) according to DIN EN ISO 11885-09. For the present study, these data were subjected to a new statistical (linear mixed models) analysis, corresponding to that used for the leaf data, in order to identify the effect of distance from the Innerste Reservoir on heavy metal levels in the floodplain soils. The floodplain was defined based on the flood hazard areas established by the NLWKN ( 2014) and covers the areas that are statistically flooded at least once every 100 years (HQextreme). At each soil sampling site, between two and four profiles were dug to a depth of 65‒145 cm and at a distance of 2‒200 m from the river. Originally, samples from four to five different horizons had been separately analyzed for each soil profile. For the present analysis, average concentrations (± standard error, SE) were calculated from these data for each profile (Fig. 2). One profile (OTH11 in Germershausen 2013) was excluded from the statistical analysis because of potential anthropogenic disturbance.

**Table 1:** Heavy metal concentrations (mg/kg dry weight) in soil samples from eleven sites along the floodplain of the Innerste River

Site	Mean distance^a^	Mean ± SD					
		Cd	Pb	Zn	Cu	Ni	Cr
LAN	0.7 km	23.88 ± 5.11	11,075.00 ± 1503.09	6875.00 ± 949.81	592.50 ± 136.36	25.63 ± 1.77	11.48 ± 1.97
PAL	8.7 km	25.81 ± 22.23	6296.67 ± 4276.28	5751.11 ± 4008.02	330.83 ± 169.77	31.28 ± 6.46	21.83 ± 9.68
OTH	13.4 km	28.50 ± 15.18	7012.50 ± 3355.78	5987.50 ± 1453.51	375.00 ± 176.96	33.38 ± 7.37	13.92 ± 26.63
BAD	28.4 km	26.00 ± 7.46	9133.33 ± 3508.78	5375.00 ± 1642.13	332.50 ± 132.61	25.42 ± 4.21	15.60 ± 5.49
GRA	35.3 km	19.43 ± 8.16	8222.00 ± 4255.31	4820.00 ± 1857.11	225.27 ± 79.32	30.60 ± 9.07	20.33 ± 9.37
HEI	43.1 km	20.06 ± 8.23	6671.18 ± 2835.05	4282.35 ± 1819.77	207.29 ± 105.95	25.12 ± 6.43	16.02 ± 6.73
ROT	50.7 km	7.21 ± 3.35	2780.67 ± 1406.25	1698.00 ± 796.63	74.73 ± 35.75	24.33 ± 7.24	23.60 ± 8.86
STE	56.7 km	7.39 ± 6.70	1928.94 ± 2024.80	1430.00 ± 1403.75	75.63 ± 61.08	23.06 ± 5.90	26.56 ± 8.80
HAS	59.6 km	7.30 ± 4.01	2748.17 ± 1865.29	1767.50 ± 922.66	86.25 ± 36.38	28.00 ± 6.50	30.17 ± 10.40
AHR	65.7 km	4.59 ± 5.48	912.31 ± 980.84	849.63 ± 896.59	44.00 ± 30.91	24.19 ± 4.43	27.44 ± 7.68
SAR	68.5 km	5.35 ± 6.15	1784.33 ± 2532.91	1208.75 ± 1403.33	57.42 ± 47.98	27.50 ± 4.03	31.50 ± 11.39
Precautionary limits for soils in Germany (mg/kg d. w.)^b^		1	70	150	40	50	60
World soil average (mg/kg)^c^		0.41	27	70	38.90	29	59.50

^a^Mean distance of soil sampling sites from the outflow of the Innerste River from the Innerste Reservoir

^b^BBodSchV ( 1999)

^c^Kabata-Pendias (2011)

#### Blackberry leaves

Between January 8 and January 23, 2020, we collected leaves of wild blackberries at five sites that were situated relatively close to five of the eleven sites from which the soil samples had previously been obtained. A total of 92 leaf samples were collected at the five sampling sites along the Innerste River, and for each site, leaves of plants growing within and outside the floodplain were obtained (Table 2). The five plant sampling sites differ with respect to the dominant type of land use:

1. Langelsheim (LAN): upstream, large settlement areas, several industrial (including metal processing) plants, and sewage treatment plant “Innerstetal”
2. Baddeckenstedt (BAD): midstream, mainly farmland
3. Heinde (HEI): lower midstream, mixture of agricultural and settlement areas
4. Hasede (HAS): downstream, nature reserve “Haseder Busch” (a hardwood alluvial forest with oaks and ashes) and sewage treatment plant “Stadtentwässerung Hildesheim”
5. Ahrbergen (AHR): lower downstream, exclusively agricultural area

**Table 2:** Heavy metal concentrations (mg/kg dry weight) in the leaves of wild blackberry from within (F) and outside (NF) the floodplain at the five study sites along the Innerste River

Area		Mean distance^a^	*n*	Metal								
				Mean ± SD	Median	Range	Mean ± SD	Median	Range	Mean ± SD	Median	Range
				Cd			Pb			Zn		
LAN	-F	1.1 km	10	0.53 ± 0.29	0.46	0.25–1.20	2.87 ± 2.72	1.85	< LOQ–9.80	125.60 ± 54.47	117.50	58.00–212.00
	-NF	0.9 km	10	0.22 ± 0.12	0.22	< LOQ–0.49	5.70 ± 2.41	5.75	2.60–10.00	41.30 ± 11.67	39.50	27.00–61.00
BAD	-F	30.7 km	6	0.66 ± 0.57	0.48	< LOQ–1.50	3.07 ± 1.20	2.70	1.90–5.20	50.00 ± 18.33	42.50	36.00–85.00
	-NF	31.3 km	5	0.25 ± 0.23	0.18	< LOQ–0.51	0.81 ± 0.64^b^	< LOQ	< LOQ–1.80	53.80 ± 24.88	41.00	37.00–97.00
HEI	-F	44.9 km	10	0.24 ± 0.17	0.19	< LOQ–0.54	1.12 ± 0.89	< LOQ	< LOQ–3.30	35.90 ± 6.52	35.50	28.00–52.00
	-NF	44.8 km	10	0.08 ± 0.05^b^	< LOQ	< LOQ–0.19	1.54 ± 3.68	< LOQ	< LOQ–12.00	31.60 ± 11.51	29.00	20.00–54.00
HAS	-F	59.5 km	11	0.38 ± 0.27	0.28	< LOQ–0.99	2.04 ± 1.86	1.50	< LOQ–6.40	56.45 ± 22.98	52.00	23.00–99.00
	-NF	59.2 km	10	0.08 ± 0.05^b^	< LOQ	< LOQ–0.17	0.71 ± 0.21^b^	< LOQ	< LOQ	41.80 ± 9.89	43.50	26.00–56.00
AHR	-F	65.0 km	10	0.26 ± 0.19	0.24	< LOQ–0.67	6.10 ± 8.58	3.65	1.00–30.00	48.80 ± 24.41	43.50	25.00–102.00
	-NF	63.4 km	10	0.06 ± 0.03^b^	< LOQ	< LOQ	1.19 ± 1.08	1.00	< LOQ–4.00	28.90 ± 4.72	29.50	21.00–36.00
				Cu			Ni					
LAN	-F	1.1 km	10	9.11 ± 1.00	8.95	7.20–11.00	1.95 ± 1.63	1.45	< LOQ–5.60			
	-NF	0.9 km	10	8.34 ± 2.09	7.70	6.20–12.00	1.92 ± 0.53	1.80	1.20–2.90			
BAD	-F	30.7 km	6	8.57 ± 3.51	7.85	5.10–14.00	0.51 ± 0.30^b^	< LOQ	< LOQ			
	-NF	31.3 km	5	8.58 ± 1.29	8.60	7.10–10.00	1.89 ± 1.20	1.80	< LOQ–3.00			
HEI	-F	44.9 km	10	8.01 ± 2.27	7.40	5.70–12.00	0.52 ± 0.36^b^	< LOQ	< LOQ–1.10			
	-NF	44.8 km	10	8.31 ± 3.69	7.00	6.20–18.00	2.23 ± 1.62	2.50	< LOQ–5.00			
HAS	-F	59.5 km	11	9.34 ± 2.18	9.50	6.30–14.00	0.74 ± 0.48	< LOQ	< LOQ–1.50			
	-NF	59.2 km	10	7.91 ± 1.63	7.95	6.10–11.00	1.77 ± 1.48	1.65	< LOQ–4.10			
AHR	-F	65.0 km	10	10.13 ± 1.32	11.00	7.30–11.00	0.58 ± 0.47^b^	< LOQ	< LOQ–1.20			
	-NF	63.4 km	10	9.75 ± 1.20	9.40	8.60–13.00	0.90 ± 0.57^b^	< LOQ	< LOQ–1.90			

^a^Mean distance of plant sampling site from the outflow of the Innerste River from the Innerste Reservoir

^b^Values are below the LOQ and should only be considered as a calculated value (not absolute). LOQs were 1.0 mg/kg for Cr, Cu, Ni, Pb, and Zn, and 0.10 mg/kg for Cd, respectively

Leaves (30 to 50 g per shrub) from 5 to 11 wild blackberry shrubs that exhibited a similar stage of development were obtained from each sampling site. To ensure independence of individual plants, the sampled shrubs within each site were situated at least 100 m apart or located on opposing sides of the riverbank. To remove external contamination by soil and dust particles, the sampled leaves were thoroughly washed with tap water for 3 to 4 min and subsequently rinsed with distilled water. They were then oven-dried (Memmert UNE 500) to constant weight at 80 °C and stored in plastic bags at − 20 °C until further analysis.

Heavy metal concentrations in the leaves were determined by a certified external laboratory (GBA Gesellschaft für Bioanalytik mbH, Hildesheim, Germany). Approximately 1 g of dried leaves per sample was digested in aqua regia (DIN EN 13657:2003–01) and analyzed by inductively coupled plasma mass spectrometry (Agilent ICP-MS 7800 and Agilent ICP-MS 7700x) according to the DIN EN 16171:2017–01 procedure. Multielement standard IV (Merck, Darmstadt, Germany) was used as the stock solution for calibration. Heavy metal determination was validated with the certified reference material “Aqua Regia Extractable Trace Elements in Soil” (BAM-U115) and “Trace Metals in Drinking Water Solution A” (CRM-TMDW-a). Limits of quantification (LOQ) were 1.0 mg/kg for Cr, Cu, Ni, Pb, and Zn, and 0.10 mg/kg for Cd. For calculations, analytical results below the LOQ were assigned a randomly generated value between zero and the LOQ. As most of the analytical results for Cr were below the LOQ, these data were excluded from statistical analyses. Heavy metal concentrations in the samples are expressed as mg/kg dry weight.

### Statistical analysis

All statistical analyses were conducted using the software program R version 4.0.4 (R Core Team 2021). Heavy metal content in floodplain soils along the river was analyzed with linear mixed models, separately for each metal, using the package “lme4” (Bates et al. 2020). The models included distance from the Innerste Reservoir as fixed effect, while site as a random factor accounted for non-independence of the 2 to 4 soil profiles per site. All heavy metal values were square-root-transformed prior to analyses to approximate homoscedasticity and normality of residuals. Significance was assessed using Wald II *F* tests with Kenward-Roger approximated degrees of freedom in the package “car” (Fox et al. 2020).

Heavy metal contents in wild blackberry leaves were analyzed with similar linear mixed models to test whether they differed between locations within and outside the floodplain and with distance from the Innerste Reservoir. The models included as fixed effects the factorial variable flood type (flooded, non-flooded), the distance of the sampling sites from the Innerste Reservoir (continuous), and the interaction of the two variables. Plant sampling sites were fitted as a random effect in all models, accounting for the non-independence of the blackberry individuals from the same sampling site. All heavy metal values were log10-transformed prior to analyses to approximate homoscedasticity and normality of residuals. Significance of the fixed effects was evaluated using a Wald II *F* test with Kenward-Roger approximated degrees of freedom as above.

Correlations among the concentrations of the different heavy metals in (a) the soil samples and (b) the blackberry leaves were tested by calculating Spearman rank coefficients using the package “corrplot” (Wei et al. 2017). To account for multiple testing, *P* values were adjusted according to the Holm-Bonferroni method, and (adjusted) *P* values < 0.05 were considered statistically significant.

## Results

### Heavy metals in floodplain soils

Average metal concentrations for the eleven soil sampling sites are given in Table 1. Mean concentrations of the six heavy metals decreased in the following order (ranges of means): Pb (912.31‒11,075 mg/kg) > Zn (849.63‒6875 mg/kg) > Cu (44‒592 mg/kg) > Ni (23.06‒33.38 mg/kg) > Cr (11.48‒31.5 mg/kg) > Cd (4.59‒28.5 mg/kg).

The statistical models indicated that the distance from the reservoir significantly influenced the soil concentrations of all heavy metals except Ni. The highest concentrations of Cd, Pb, Zn, and Cu occurred at a short distance from the Innerste Reservoir, and the levels of these four metals decreased downstream (Table 3 and Fig. 2). In contrast, Cr concentrations in the floodplain soils increased downstream.

**Table 3:** The effect of distance from the Innerste Reservoir on heavy metal content in soils. Results are based on linear mixed effects models with Wald II *F* tests with Kenward-Roger approximated residual degrees of freedom.* P* values < 0.05 are given in bold

	Distance	
	*F*_1,9.4–9.7_	*P* value
Cd	37.39	0.00013***
Pb	24.88	0.00065***
Zn	54.66	< 0.0001***
Cu	95.15	< 0.0001***
Ni	3.61	0.0870 (*)
Cr	8.17	0.0178*

Concentrations of Pb, Zn, Cu, and Cd in soil were strongly correlated (*rho-*values between 0.88 and 0.96, all *P* < 0.001). A moderate positive correlation existed between Cr and Ni values (*rho* = 0.58, *P* < 0.001). Moderate negative correlations were found for Cr and Pb (*rho* =  *− *0.57, *P* < 0.001) as well as for the relationships between Cr and Cd, Cu, and Zn, respectively (all *rho* =  *− *0.47, all *P* < 0.001) (Fig. 4A).

### Heavy metals in blackberry leaves

**Fig. 3:**
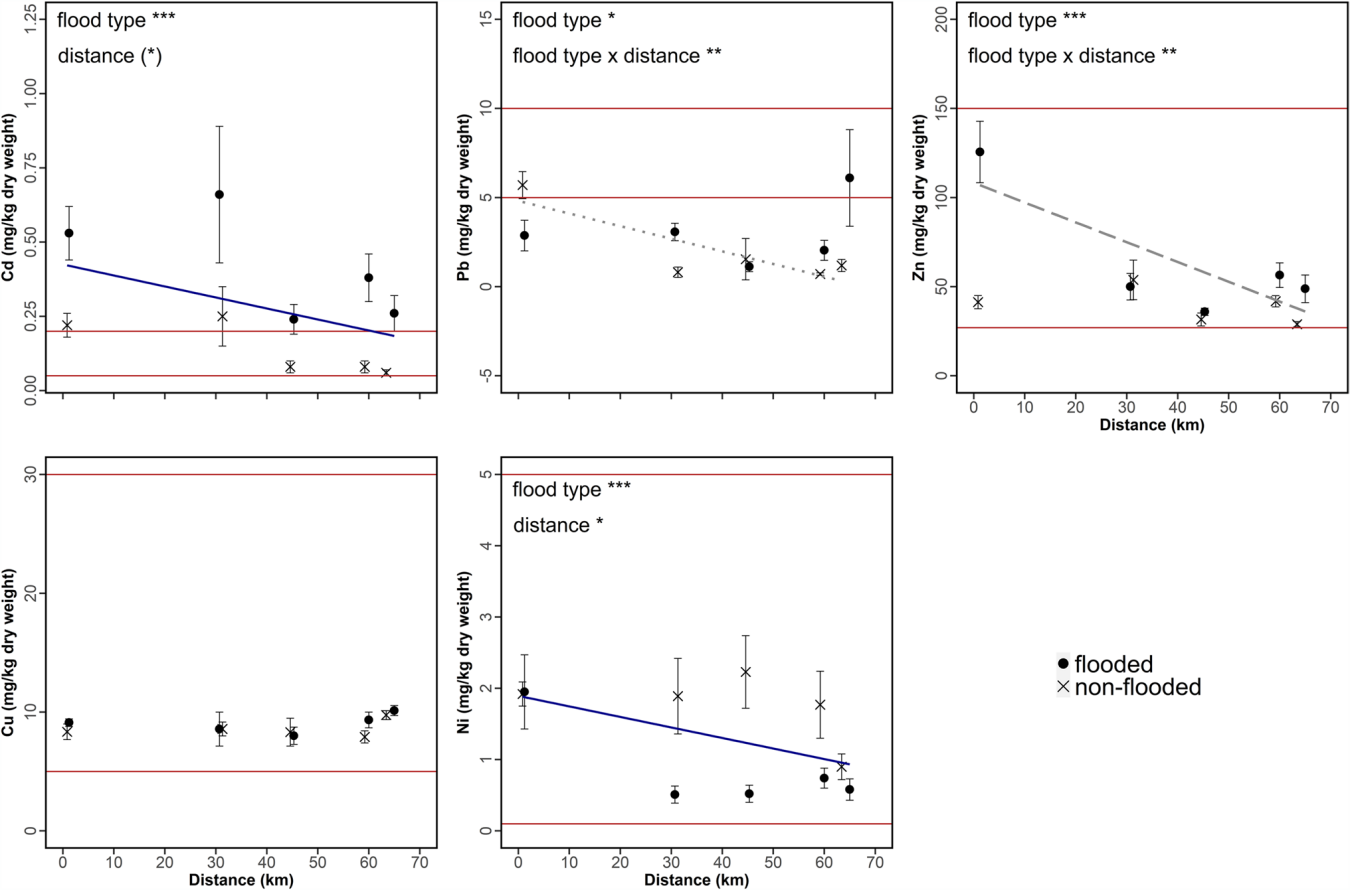
Mean (± SE) heavy metal concentration in leaves of 92 wild blackberry plants, sampled within and outside the floodplain (factor flood type; flooded vs. non-flooded area) at five sampling sites along the Innerste River with increasing distance from the Innerste Reservoir. The blue line represents a common slope for both flood types, while the dotted (non-flooded) and long-dashed (flooded) lines show slopes depending on the flood type. The horizontal red lines represent the normal range for mature plant leaves according to Kabata-Pendias (2011). Significant effects are indicated for ease of interpretation (see Table 4 for full statistical results). Note the different scaling for each heavy metal. (*) 0.05 < *P* < 0.1, * *P* < 0.05, ** *P* < 0.01, *** *P* < 0.001

The concentrations of the five heavy metals in leaves of wild blackberries from the five plant sampling sites along the Innerste River are presented in Table 2 and Fig. 3. In both flooded and non-flooded areas, mean heavy metal concentration in the leaves decreased in the following order (ranges of means, flooded; non-flooded): Zn (35.9‒125.6; 28.9‒53.8 mg/kg) > Cu (8.01‒10.13; 7.91‒9.75 mg/kg) > Pb (1.12‒6.1; < 1.0‒5.7 mg/kg) > Ni (< 1.0‒1.95 mg; < 1.0‒2.23 mg/kg) > Cd (0.24‒0.66; < 0.1‒0.25 mg/kg). At all sites, mean Cd values for leaves from blackberries growing within the floodplain exceeded the range of normal values for uncontaminated mature plant leaves given by Kabata-Pendias (2011). Mean Cu and Ni contents in the blackberry leaves were within the normal range of values at all sampling sites within and outside the floodplain (Table 5 and Fig. 3), while mean Pb levels were below the normal range, except for the non-flooded area of LAN (5.7 ± 2.41 mg/kg) and the floodplain area of AHR (6.10 ± 8.58 mg/kg). For Zn, the vast majority of leaf concentrations were within the normal range given by Kabata-Pendias (2011); however, values measured for leaves from the flooded area of LAN were at the threshold of toxicity (Table 2 and Table 5).

The statistical models showed that flood type significantly affected the Cd, Pb, Zn, and Ni concentrations of the leaves (Table 4), with overall higher Cd, Pb, and Zn values for plants from within and higher Ni values for those from outside the floodplain. However, near the Innerste Reservoir, Pb concentrations in leaves were higher in blackberries from the non-flooded than the flooded area (Fig. 3). The model further revealed that the concentrations in the wild blackberry leaves differed marginally significantly with distance from the Innerste Reservoir for Cd, and significantly for Ni (Table 4), with higher levels occurring at shorter distance from the reservoir. The model also indicated that the interaction of flood type and distance from the reservoir affected leaf concentrations of Pb outside the floodplain and Zn concentration of leaves inside the floodplain (Fig. 3 and Table 4).

**Table 4:** The effects of flood type (flooded and non-flooded) and distance from the Innerste Reservoir on heavy metal content in wild blackberry leaves. Results are based on linear mixed effects models with Wald II *F* tests with Kenward-Roger approximated residual degrees of freedom. *P *values < 0.05 are given in bold

	Flood type		Distance		Flood type × distance	
	*F*_1,85_	*P* value	*F*_1,2.5–2.9_	*P* value	*F*_1,85_	*P* value
Cd	52.90	< 0.0001***	8.31	0.0716 (*)	0.49	0.485^ ns^
Pb	6.88	0.0103*	1.46	0.316^ ns^	10.91	0.0014**
Zn	31.68	< 0.0001***	4.81	0.12^ ns^	10.86	0.0013**
Cu	1.50	0.225^ ns^	0.45	0.55^ ns^	0.00	0.975^ ns^
Ni	16.62	0.0001***	13.37	0.0488*	0.38	0.540^ ns^

**Fig. 4:**
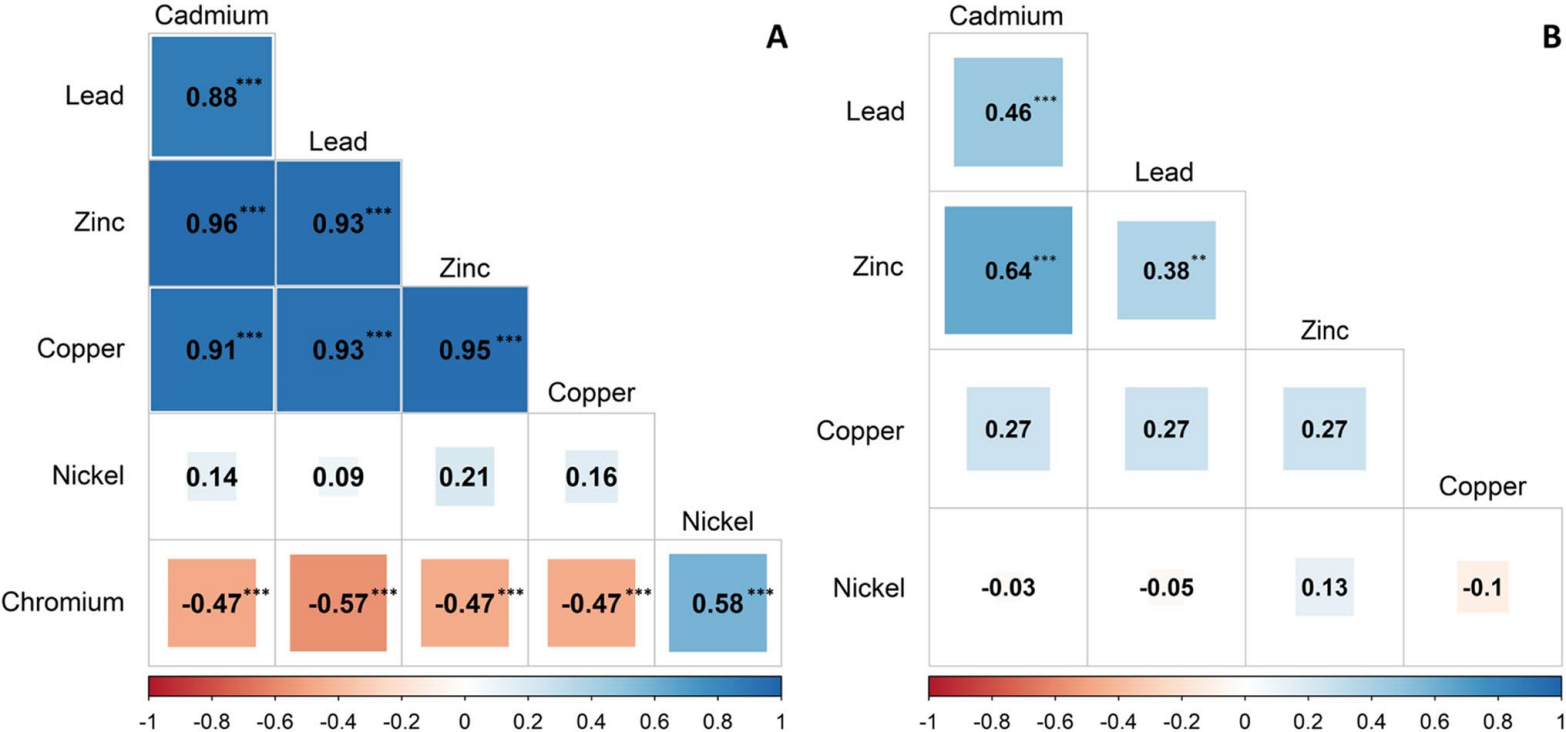
**A** Spearman’s correlation matrix of total heavy metal concentrations among 36 soil samples. **B** Spearman’s correlation matrix of total heavy metal concentrations among leaves from 92 blackberry plants. The size of the boxes indicates the strength of the relationship and the color gradient from red to blue shows the change of the relationship from negative to positive. *P* values are Bonferroni adjusted. ** *P* < 0.01, *** *P *< 0.001

Leaf concentrations of Zn and Cd (*rho* = 0.64, *P* < 0.001), Pb and Cd (*rho* = 0.46, *P* < 0.001), and Pb and Zn (*rho* = 0.38, *P* < 0.01) were positively correlated (Fig. 4B).

## Discussion

### Heavy metals in floodplain soils

The present study revealed that the floodplain soils along the Innerste River are heavily contaminated with the heavy metals Cd, Pb, Zn, and Cu that are typical constituents of mill tailings and slag wastes from the historic ore mining, processing, and smelting in the Harz Mountains. At all sampling sites, mean soil levels of Pb, Zn, Cu, and Cd markedly exceeded the precautionary limits of the German Federal Soil Protection and Contaminated Sites Ordinance (BBodSchV, 1999) and were also considerably higher than the global average values for soils given by Kabata-Pendias (2011). Concentrations of Ni and Cr in the Innerste floodplain did not exceed the precautionary limits for soils stipulated by federal regulations in Germany (BBodSchV, 1999). Except for Ni at sites PAL, OTH, and GRA, all Ni and Cr values were below the global average for soils (Table 1 and Fig. 2). Our results are in line with those of previous studies (Nowak and Preul 1971; Hellwig 2002; Knolle et al. 2011) and demonstrate the lasting impact of the former mining activities in the Harz Mountains, as well as the crucial role of riverine ecosystems in the dispersal of the heavy metals. The massive heavy metal contamination of the Innerste floodplain was recently again confirmed for lead and zinc at the downstream site of Ahrbergen (Steingräber et al., unpublished observations).

The Innerste Reservoir acts as an artificial sediment trap and can thus be considered a sink for heavy metals, corresponding to the situation described for other artificial reservoirs in mining areas (García-Ordiales et al. 2016). It has previously been concluded that the larger part of the heavy metal load present in the river sediments and the floodplain soils downstream of the Innerste Reservoir reached the area prior to its construction (Ernst et al. 2009).

Concentrations of Cd, Pb, Zn, and Cu in floodplain soils decreased significantly with distance from the Innerste Reservoir. The decrease of heavy metal concentrations along the course of the Innerste River is typical for floodplain soils (Hilscherova et al. 2007; Hürkamp et al. 2009). The concordant decrease in Cd, Pb, Zn, and Cu contents with distance from the Innerste Reservoir and the positive correlations for the concentrations of these heavy metals indicate that the metals in the floodplain soils originate from the same source (Abderahman and Abu-Rukah 2006). The heavy metals in the floodplain soils of the Innerste River were washed out from waste heaps (Meyer 1822; Hellwig 2002; Knolle 2009; Germershausen 2013). As in other contaminated river systems (Hilscherova et al. 2007; Hürkamp et al. 2009; Schulz-Zunkel and Krueger 2009), it is assumed that the heavy metal-laden sediments of the Innerste River were transported downstream and repeatedly redistributed during flooding events and intense rainfall (Du Laing et al. 2009; Ponting et al. 2021). According to Fan et al. (2021), mining and other industrial activities in upstream areas will result in higher heavy metal concentrations compared to downstream areas. Lower metal levels in the latter can be attributed to dilution effects from inflow of unpolluted waters (Luo et al. 2020). This is probably also a main factor explaining the decrease of metal levels (except Cr) in floodplain soils with distance from the Innerste Reservoir.

The mobility of heavy metals in soils depends on chemical, physical, and biological factors, including soil texture (proportion of clay minerals), pH, organic matter, salinity, redox potential, temperature, soil organisms, and vegetation (Du Laing et al. 2009; Schulz-Zunkel and Krueger 2009; Hu et al. 2017; Ponting et al. 2021). It has been shown that periodic flooding of floodplain soils affects a number of these factors, especially pH and redox potential (Du Laing et al. 2009; Schulz-Zunkel and Krueger 2009). Further studies addressing these issues in the Innerste floodplain are recommended.

Nickel and Cr are not associated with mining in the Harz Mountains and therefore probably derived from other sources. Major anthropogenic entry routes of Cr are wastewater, sewage sludge, and mineral fertilizers (Gonnelli and Renella 2013; Stückrad and Wilcke 2013,2013), while mineral fertilizer, manure, and sewage sludge constitute the most important sources of Ni in agricultural areas (Gonnelli and Renella 2013; Wilcke 2013). This is also considered to be the case in our study area.

### Heavy metals in blackberry leaves

Overall, leaves of wild blackberry plants growing in the heavily metal-contaminated soils of the Innerste floodplain showed significantly higher concentrations of Cd, Pb, and Zn compared to those from outside the floodplain (Table 4). Similar findings were reported by Madejón et al. (2004) in a study on *Populus alba* in Spain. The levels of Cd, Zn, and Ni in the leaves decreased with distance from the Innerste Reservoir (Fig. 2 and Fig. 3). This matches the findings for the floodplain soils and suggests that variation in leaf concentrations reflects those in the soils (Parzych and Sobisz 2018). Given the long-standing nature of the heavy metal contamination of the Innerste floodplain, this conclusion seems justified, even though the soil and leaf samples were obtained during different years. An unexpected finding was the higher concentration of Pb in leaves of blackberries from outside compared to those growing within the floodplain close to the Innerste Reservoir near Langelsheim (Fig. 3). We suspect that this may reflect Pb exposure from mine tailings and slag waste deposited outside the floodplain or to excavated river sediment dumped during construction of the reservoir.

Uptake and accumulation of heavy metals by *R. fruticosus* from contaminated and uncontaminated sites were previously studied by different authors (Dorrington and Pyatt 1983; Yoon et al. 2006; Alagić et al. 2016; Nujkić et al. 2016; Lassalle et al. 2021). It has been demonstrated that blackberries growing on contaminated soils accumulate higher amounts of heavy metals (Alagić et al. 2016; Nujkić et al. 2016). For comparison with our data, Table 5 lists concentrations in blackberry leaves that were reported by two other studies (Alagić et al. 2016; Lassalle et al. 2021).

**Table 5:** Heavy metal concentrations (mg/kg) in blackberry leaves from the Innerste study region compared to data from two other studies (means ± SDs), typical and toxic concentrations in plant leaves, and maximum tolerable levels (MTL) in animal feed

	Blackberry leaves (this study)		Blackberry leaves (brownfield, exact study site not given)^a^	Blackberry leaves (Minićevo, Serbia)^b^	Sufficient or normal content in mature leaves^c^	Excessive or toxic content in mature leaves^c^	MTL in feed for livestock^d^
	Within floodplain	Outside floodplain					
Cd	0.39 ± 0.32	0.13 ± 0.12	-	0.17 ± 0.03	0.05–0.2	5–30	10
Pb	3.01 ± 4.47	2.12 ± 2.84	-	1.16 ± 0.36	5–10	30–300	10–100
Zn	64.34 ± 44.08	37.89 ± 14.07	86.91 ± 24.27	20.24 ± 4.52	27–150	100–400	300–1000
Cu	9.08 ± 2.11	8.58 ± 2.26	17.44 ± 5.91	12.46 ± 2.58	5–30	20–100	15–250
Ni	0.89 ± 0.98	1.72 ± 1.21	1.71 ± 0.44	2.64 ± 0.57	0.1–5	10–100	50–250

^a^Lassalle et al. ( 2021)

^b^Alagić et al. ( 2016)

^c^Kabata-Pendias ( 2011)

^d^National Research Council ( 2005)

Metal uptake by plant roots occurs either passively with water uptake or actively through transport mechanisms across the plasma membrane of the rhizodermis (Yoon et al. 2006; Tangahu et al. 2011). Essential metals (such as Cu, Ni, and Zn) are subject to physiological regulation and their uptake is selective, while that of nonessential elements (such as Cd and Pb) is not (Du Laing et al. 2009; Kabata-Pendias 2011; Salinitro et al. 2019). Zinc uptake by plants increases linearly with its concentration in soil (Madejón et al. 2004; Kabata-Pendias 2011). The concentrations of Cu in the blackberry leaves from within and outside the floodplain were not significantly different, which is consistent with the view that transfer of Cu to aboveground parts is limited and concentration in aboveground plant parts is constant over a wide range of soil Cu contents (Parzych and Sobisz 2018). In contrast, nickel concentrations in wild blackberry leaves outside the floodplain were significantly elevated compared to those within the floodplain. It is hypothesized that this mainly reflects the application of mineral fertilizers, manure, and sewage sludge in the former areas.

In terms of food web transfer to herbivores, overall mean leaf concentrations were below the maximum tolerable levels (MTLs) specified by the National Research Council (2005) for livestock feed (Table 5). A few Pb values of leaves sampled within the floodplain at Ahrbergen exceeded levels associated with livestock toxicity. Therefore, the possibility of excess exposure of herbivores must be taken into account (Reglero et al. 2008), especially considering additional uptake of dust and grit attached to plant surfaces (Vlad et al. 2019). In fact, Pb toxicosis has been repeatedly reported in livestock grazing in the Innerste floodplain (Meyer 1822; Haarstick 1910; Knolle and Knolle 1983; Knolle et al. 2011).

## Conclusions and outlook

The floodplain soils of the Innerste River are highly contaminated with Cd, Pb, Zn, and Cu due to historical metal ore mining in the Harz Mountains and related ore processing and smelting activities. Except for Cr, heavy metal concentrations in the floodplain soils decreased downstream. Levels of Cd, Pb, and Zn in blackberry leaves sampled within the floodplain typically exceeded concentrations in leaves sampled outside the floodplain. For Ni, higher values were measured in leaves of plants from outside the floodplain. Notwithstanding the fact that the soil and plant data were not obtained at the same time, the results of the present study suggest that heavy metal levels in soil affect those in the leaves of wild blackberries growing on these soils and, in the case of Cd, Pb, Zn, and Ni, reflect the difference between the flooded and non-flooded areas. A study addressing soil-root-leaf transfer of metals (Pb, Zn) is currently undertaken that will enhance our understanding of the physiological mechanisms underlying metal uptake by blackberries and their potential as a biomonitor of heavy metal pollution in the Innerste floodplain.

## Data Availability

The datasets used and analyzed during the current study are available from the corresponding author on reasonable request.
